# Characteristics of successful government-led interventions to support healthier populations: a starting portfolio of positive outlier examples

**DOI:** 10.1136/bmjgh-2023-011683

**Published:** 2023-05-23

**Authors:** Peter Bragge, Alex Waddell, Paul Kellner, Veronica Delafosse, Robert Marten, Anders Nordström, Sandro Demaio

**Affiliations:** 1 Monash Sustainable Development Institute Evidence Review Service, Monash University, Clayton, Victoria, Australia; 2 Monash University, Clayton, Victoria, Australia; 3 World Health Organization, Geneva, Switzerland; 4 Government Offices of Sweden, Stockholm, Sweden; 5 Victorian Health Promotion Foundation, Carlton South, Victoria, Australia

**Keywords:** health policy, health services research, other study design, public health

## Abstract

Despite progress on the Millennium and Sustainable Development Goals, significant public health challenges remain to address communicable and non-communicable diseases and health inequities. The Healthier Societies for Healthy Populations initiative convened by WHO’s Alliance for Health Policy and Systems Research; the Government of Sweden; and the Wellcome Trust aims to address these complex challenges. One starting point is to build understanding of the characteristics of successful government-led interventions to support healthier populations. To this end, this project explored five purposefully sampled, successful public health initiatives: front-of-package warnings on food labels containing high sugar, sodium or saturated fat (Chile); healthy food initiatives (trans fats, calorie labelling, cap on beverage size; New York); the alcohol sales and transport ban during COVID-19 (South Africa); the Vision Zero road safety initiative (Sweden) and establishment of the Thai Health Promotion Foundation. For each initiative a qualitative, semistructured one-on-one interview with a key leader was conducted, supplemented by a rapid literature scan with input from an information specialist. Thematic analysis of the five interviews and 169 relevant studies across the five examples identified facilitators of success including political leadership, public education, multifaceted approaches, stable funding and planning for opposition. Barriers included industry opposition, the complex nature of public health challenges and poor interagency and multisector co-ordination. Further examples building on this global portfolio will deepen understanding of success factors or failures over time in this critical area.

**WHAT IS ALREADY KNOWN ON THIS TOPIC**

- Addressing global public health challenges remains a complex challenge for governments around the world.
- This has led to calls to strengthen global networking and knowledge sharing to optimise efforts to promote healthy populations.

**WHAT THIS STUDY ADDS**

- This study drew on rapid evidence and practice review principles to bring together five ‘mini-reviews’ (Google Scholar search and one semistructured interview) of successful government-led interventions to promote healthier populations spanning a broad range of settings and topics.
- Facilitators of success across the examples included political leadership, public education, multifaceted approaches, stable funding and planning for opposition.
- Barriers included industry opposition and poor interagency and multisector co-ordination.

**HOW THIS STUDY MIGHT AFFECT RESEARCH, PRACTICE OR POLICY**

- This starting portfolio of five examples outlines a novel, reproducible approach designed to facilitate further examples with minimal resource cost.
- Adding to this portfolio would build a rich picture of key factors aiding or hindering public health efforts around the world.
- Governments, practitioners and researchers can use this information to optimise investment and reduce risk in addressing this critical area of ongoing need.

## Introduction

Addressing global public health challenges has been a major focus of the United Nations Millennium Development Goals (MDGs) from 2000 to 2015^1^ and subsequently the current Sustainable Development Goals.^2^ The 2015 MDG report highlighted major progress—for example, halving of the child mortality rate and a 45% drop in maternal mortality rate between 1990 and 2015.^3^ However, significant challenges remain. The COVID-19 pandemic contributed to 15 million global deaths in 2020 and 2021; triggered a substantial rise in mental illness; stymied progress on universal health coverage; hampered progress against HIV, tuberculosis and malaria; and severely impacted the health workforce. Regional disparities on maternal and child health remain; more children are missing essential vaccines for other diseases^4^; and non-communicable diseases caused by tobacco, alcohol, poor diet, lack of physical exercise and air pollution are compounding these COVID-19 and other health burdens.^5^ Threats and challenges with the rapidly changing climate are also increasingly threatening human and planetary health.^6^

To address these complex, intersecting challenges the WHO’s Alliance for Health Policy and Systems Research; the Government of Sweden; and the Wellcome Trust formed the Healthier Societies for Healthy Populations initiative in February 2020. The initiative aims to develop a global systems and policy research agenda to underpin efforts to promote healthy populations through global networking and codesign.^5^ This effort harnesses opportunities unlocked by the COVID-19 pandemic for governments to work creatively to optimise global health beyond the avoidance and management of major health emergencies.^7^

Advancement of the Healthier Societies for Healthy Populations agenda will be optimised if success factors or failures across a broad array of policy processes can be better understood. As a first step towards building such understanding, the group commissioned a rapid review with the primary aim of exploring characteristics of successful or less successful primarily government-led interventions to support healthier populations.

## Methods

The review drew on established review methodologies. Rapid reviews are a form of knowledge synthesis accelerating traditional systematic review processes by streamlining or omitting some review tasks.^8^ Examples include focusing on particular types of evidence (eg, only evaluating reviews rather than primary studies, or not including grey literature); searching a smaller number of academic databases; and limiting the time period.^9^ Evidence and practice reviews combine rapid or systematic reviews with information from a small number of one-on-one interviews or focus groups. The purpose of the interviews is not to provide a comprehensive qualitative exploration, but to supplement and contextualise global published knowledge in specific contexts or settings.^10^ Such practice exploration can provide vital insights.^11 12^

We adapted these established methods to address the specific remit of this review. A key challenge was that ‘healthy populations’ needed to be defined broadly because the scope of Healthier Societies for Healthy Populations not limited to a specific health, population or other domain. Given a desktop rapid review was unfeasible even using rapid review approaches, we conducted a series of ‘mini-reviews’ focusing on ‘outlier examples’—which we define for the purpose of this review as government-led interventions to promote healthier populations which have documented evidence of successful outcomes. These ‘outlier examples’ were purposefully sampled based on deliberations by the Healthier Societies for Healthy Populations group. Specifically, authors on this paper who were also part of the Healthier Societies for Healthy Populations group (AN, RM and SD) shortlisted a series of candidates based on their knowledge and the deliberations from the Healthier Societies meetings. The author team then deliberated on a selection that aimed to represent different geographical regions and include high-income and low-income/middle-income countries. Our final selection of five examples reflected these considerations as well as key population health issues identified by the Healthy Societies group—tobacco and alcohol use, obesity and preventable injuries (5):

1. Front-of-package warnings on food labels containing high sugar, sodium or saturated fat (Chile).^13^
2. Multifaceted healthy food initiative
  - Restriction on the use of trans fats.
  - Calorie labelling in food service outlets.
  - Cap on the size of sugary beverages sold (New York).^14^
3. Alcohol sales and transport ban during COVID-19 (South Africa).^15^
4. Vision Zero road safety initiative (Sweden).^16^
5. Establishment of the Thai Health Promotion Foundation (Thailand).^17^

Given the examples included those from low-income and middle-income countries an author reflexivity statement was completed (online supplemental file 1).^18^ For each example, a single semistructured one-on-one interview was undertaken by a member of the research team (PB, AW and PK) with a person with leadership, in-depth knowledge and/or deep experience of the intervention. The interview framework (online supplemental file 1) focused on gathering high-level reflections on success factors for the intervention and barriers to implementation. It was not appropriate or possible to involve patients or the public in the design, or conduct, or reporting, or dissemination plans of our research.

10.1136/bmjgh-2023-011683.supp1 Supplementary data

For the literature scan a specialist librarian developed a Google Scholar search string pertaining to each initiative, informed by relevant studies already known and/or supplied by the interviewee (online supplemental file 2). The researcher who conducted the interview screened the first 50 Google Scholar results, ordered by relevance. Eligible articles were those in English with a primary focus on at least one of four characteristics pertaining to the outlier example:

- Theory of change (how the policy intervention is postulated to work).
- Effectiveness (studies empirically testing the intervention or presenting evaluation findings).
- Spread (evidence that the intervention has seeded similar interventions elsewhere and/or is itself adopted from a previous intervention elsewhere).
- Implementation considerations (barriers, facilitators, adapting to different contexts).

Key themes for the interviews, and information pertaining to these four characteristics, were consolidated into a short report for each outlier example (online supplemental file 3). Analysis of themes across the examples focused on identifying cross-cutting themes across the broad array of interventions and settings, in particular barriers and facilitators to successful interventions. These are the focus of the results section presented below.

## Results

The five interviews were undertaken between 17 August 2022 and 31 August 2022. Interview durations ranged from 26 to 52 min, with an average duration of 36 min. Following examination of titles, abstracts and where necessary full-text references, a total of 169 studies (48%) were deemed relevant across the examples (online supplemental file 3).

### Facilitators and barriers to success of initiatives

Table 1A, B presents facilitators and barriers of successful interventions across the five examples, respectively, highlighting those identified in interviews (shaded cells) and supported by published studies (numbers within cells). These are described below with supporting quotes from the interviews (italicised text).

**Table 1:** (A) Facilitators and (B) barriers associated with outlier examples based on key themes from interview (shaded boxes) and published studies (numbered references)

	Food labels Chile	Healthy food NYC	COVID-19 ban South Africa	Vision Zero Sweden	ThaiHealth
(A) Facilitators (n interviews, n references)					
Political leadership (5, 2)				21	19
Public education (3, 3)		54 55			19
Evidence based (3, 2)	20				19
Multifaceted (3, 2)		56 57			
Addressing opposition arguments (4, 0)					
Shared goals (2, 2)	20			21	
Involving stakeholders (3, 0)					
Data insights/information systems (2, 1)		58			
Public support (1, 2)		58 59			
Multidisciplinary teams (2, 0)					
Economic case for action (2, 0)					
Shift in thinking about the problem (1, 1)				21	
Political continuity (1, 1)		60			
Independent of bureaucracy therefore able to partner with others (1, 1)					19
Broad legislative scope (1, 0)					
Common goal (1, 0)					
Positive industry response/compliance (1, 0)					
‘Invisible’ change (trans fat removed) (1, 0)					
Connection of public health with healthcare system (1, 0)					
Window of opportunity (1,0)					
Bipartisanship (1, 0)					
Presence of a similar initiative (1, 0)					
Stable funding (1, 0)					
Ability to incubate related institutions (1, 0)					
Accountability and transparency through civil society participation (1, 0)					
(B) Barriers (n interviews, n references)					
Employment and trade implications (2, 4)	23 24		25 26		
Industry opposition (4, 2)	20		22		
Poor co-ordination between agencies (2, 2)	27			28	
Freedom of choice argument (1, 3)		50 61 62			
Consumers can bypass law (1, 2)		31 32			
Associated consequences—alcohol withdrawal, illegal brewing (1, 2)			29 30		
Developing operational definitions for creating laws (1, 1)	20				
Involving multiple stakeholders (1, 1)	20				
Political changes (1, 1)	20				
Other contributing factors to the problem not addressed (1, 0)					
Multiple stakeholder groups opposed (1, 0)					
Presumes information leads to behaviour change (1, 0)					
Government inefficiencies and staff turnover (1, 0)					
Not sustainable (even when watered down)					

NYC, New York City.

### Facilitators

The most frequently identified facilitator was political leadership, which was identified in all five interviews and in two published studies—for example, ThaiHealth ‘*is governed by a board chaired by Prime Minister…and it is a multi-sectoral multi-stakeholder governing board.’* Public education and awareness campaigns were also prominent facilitators (three interviews, three published studies)—for example, the ThaiHealth initiative involved social marketing to raise awareness combined with advocacy to create a conducive environment for behaviour change.^19^ Using a multifaceted approach and drawing on an evidence base were each supported by three interviews: ‘*…in Chile, the government really has the tradition of receiving a lot of knowledge from the academia. So it’s very typical that they call experts to comment or receive advice’* (table 1A).

The ability to foresee and address opposition arguments was also seen as critical across four of the five outlier examples: ‘*I certainly didn't want to stick my head out there, so I brought in other experts and civil society people and worked very hard with the media to defend the policy’ (Alcohol ban, South Africa*). Another strategy described for dealing with opposing interests was paying careful attention to who is involved in intervention design; ‘*I don't think that industry should be, or at least big corporates should be included in the discussion of defining the goals or the limits of policies, if we are really interested in promoting health.’ (Food labelling, Chile*) Shared goals were highlighted as facilitators in the food labelling^20^ and road safety interviews,^21^ both of which were characterised by the need for multiple organisations/agencies to work collaboratively. Another key ingredient was the involvement of stakeholders to build coalitions (three interviews), illustrated by this reflection from the New York example: ‘*There are ways of doing successful advocacy. You get as many people on board as possible.’*

Less-frequently reported ingredients of success were also observed. The ‘invisibility’ of removal of trans fats in New York City was seen as a factor in its success: ‘*They went around and told restaurant people they were doing this, demonstrated that nobody could see, taste or smell the difference.’* The alcohol sales ban in South Africa took advantage of a window of opportunity presented by COVID-19, leading to dramatic reductions in alcohol-related violence, injury and death. Unlike other examples, the Vision Zero initiative was characterised by a shift in the understanding of road trauma. The degree to which legislators bought into the idea of a systems approach to road safety, as opposed to a narrower focus on individual behaviour and responsibility, was seen as pivotal to the implementation. Crucially, Vision Zero advanced a compelling proposition to encourage this shift: *“it becomes impossible to say no [to the proposition of aiming for zero road deaths], because you would be seen as cold-hearted … the minister … made it her own saying, ‘Road traffic should be as the workplace. Everyone should be expected to come home alive after a workday or in road traffic.’*

### Barriers

Of the identified barriers to success, industry opposition was a dominant theme, being represented across four of the five interviews and two supporting studies.^20 22^ Equally prominent was negative employment and trade implications, noted in the Chile^23 24^ and South Africa examples.^25 26^ Although impact on food and beverage industry profits was minimal in the Chile example,^23^ economic impacts of the South Africa alcohol bans resulted in unemployment, which was described as a factor in promoting violent behaviour^25^ and imposing negative impacts on existing inequalities in the accommodation and tourist workforce sector.^26^ For both food labelling^27^ and Vision Zero,^28^ co-ordination between sectors and industries to collectively address a system issue was a major challenge. The barrier of unintended consequences was noted across two interviews, with each supported by two published studies—the South African alcohol ban highlighted unintended consequences of alcohol withdrawal (which caused a similar ban in France to be overturned) and a rise in illegal brewing^29 30^; New York’s health food initiatives were capable of being bypassed by consumers either going into nearby jurisdictions where the initiatives were not active^31^ or by taking advantage of free refills^32^ (table 1B).

### Insights from the literature

Table 2A–D summarises key themes from literature across the key characteristics of theory of change, effectiveness, spread implementation considerations.

**Table 2:** Summary of findings from literature by theme

Initiative	Key findings from literature and supporting references
A.Theory of change	
Food labels (Chile)	Front of package (FOP) labels are postulated to work by providing consumers with additional information about contents of the food or beverage thereby encouraging them to choose the healthier option.^33^ It is also said to work at the industry level by encouraging manufacturers to improve their food and beverage items.^34^
Healthy food (New York City NYC)	Provision of information about calories will inform decision-making about food purchases.^35^ Adverse health effects of trans fats have been demonstrated.^63^ Cap on the size of sugary beverages sold changes choice architecture which means knowledge about caloric intake is less relevant;^36^ people eat more from bigger containers even if not hungry and food not palatable.^37^
COVID-19 Alcohol sales and transport ban (South Africa)	Alcohol use associated with undermining of social distancing and compromising immune response.^22 38^
Vision Zero road safety (Sweden)	Vision Zero is based on the ideas that responsibility for road safety is not limited to the actions of road users; it is also a responsibility of system operators: *“tradition and road traffic rules for the road users have been used as an excuse for not undertaking necessary system changes and modifications” (p. 2) … “It is human to make mistakes, and we must design for the human as we are, not the perfect human that in reality does not exist” … (p. 4*). System modifications, for example, airbags and road safety barriers, are designed based on strategies to *“control, harness, reduce, cushion, or redirect harmful kinetic energy”*, which was described as a key contributor to road traffic injury and death as far back as 1970.^64^ Ways of holding road users accountable through road laws such as speed and alcohol limits are well understood. The theory of change for system designers is similar, but based on formal regulations and road safety standards, for example, mandating airbags in car manufacturing.^39^
ThaiHealth (Thailand)	Social marketing is combined with strategically networked advocacy partnerships to promote both awareness-raising and behaviour change—based on the realisation that awareness alone is not sufficient for behaviour change; a conducive environment is also required.^19^
B. Effectiveness	
Food labels (Chile)	There is evidence to suggest FOP labels change food at the system level by encouraging industry through reformulation of products.^40^ There is also evidence to suggest consumer’s knowledge is changed by the use of FOP.^41^ Little evidence for the ongoing effects of FOP on consumer behaviour.^34^ In Mexico, consumers were mistrusting of the labels.^65^
Healthy food (NYC)	Some evidence that people who see calorie labels purchase less calories.^35 66^ However, awareness and impact may wane over time^67^ and interpretation of meaning of messages may be difficult.^68^ Mixed evidence re labelling and BMI^69 70^ and impact on ordering.^71 72^ Restriction on the use of trans fats led to lower hospital admissions for heart attacks.^42^ Cap on the size of sugary beverages sold appeared to lower consumption when in place.^73^
COVID-19 Alcohol sales and transport ban (South Africa)	80% decrease in rapes and aggravated assaults,^43^ halving of the unnatural death rate,^44^ reduction on trauma admissions especially violence related.^74 75^ Reduced consumption, but not in problem drinkers.^45^
Vision Zero road safety (Sweden)	‘2+1’ roads (standard 2-lane road is converted to three lanes to create an overtaking lane which alternates every few kilometres, with the two directions separated by a physical barrier)^76^ reduced risk of fatality by 80%.^77^ Lowering speed limits has been shown to reduce fatalities.^46 78^ Reduction in road deaths per 100 000 people from 6 to 4.7 in the decade following implementation of Vision Zero.^46^ Where large-scale attempts to implement design principles have been made, fatalities can be reduced by up to 90% compared with 2%–3% reduction in areas where no such improvements have been made.^46^ The Decade of Action for Road Safety 2011–2020 aimed to stabilise, then reduce the global number of road fatalities. Although global road deaths are below the 1.9 million in a ‘no action’ scenario, these aims were not met.^79^
ThaiHealth (Thailand)	Decrease in smoking prevalence from 25.47% to 19.94%; 13% decrease in alcohol consumption; alcohol free Buddhist lent period led to 20% reduction in road accidents from drunk driving; road safety return on investment 130.2 baht for each 1 baht invested.^19^
C. Spread	
Food labels (Chile)	Other countries have followed Chile’s use of FOP labels—Peru, Uruguay and Mexico; Australia uses a star image system.^47^
Healthy food (NYC)	Calorie labelling laws spread to other states, cities and countries following their introduction in NYC.^35 80^ Restriction on the use of trans fats has been adopted in >40 countries.^42^
COVID-19 Alcohol sales and transport ban (South Africa)	Other countries that instituted pandemic-associated alcohol restrictions included India, Nepal, Slovenia, India and Thailand^29^ Georgia, Greenland and Russia.^30^
Vision Zero road safety (Sweden)	Vision Zero approach has been adopted across many countries including Norway, Australia, New Zealand, Poland, UK, Germany, USA and India. However, implementation is in various stages and implementation challenges have been reported, for example, political commitment and funding.^48 81^
ThaiHealth (Thailand)	ThaiHealth has supported establishment of similar organisations in Malaysia, South Korea, Mongolia and Tonga; there is an International Network of Health Promotion Foundations further reflecting global spread—Austria, Taiwan as well as the above countries.^19^
D. Implementation considerations	
Food labels (Chile)	Villalobos Dintrans^20^ notes barriers including involving multiple stakeholders, political changes and the process undertaken to define the law (lack of legal precedent). The law faced challenges from the food and advertising industries due to potential impacts on profits. This was countered by arguments spotlighting the need for drastic changes to affect change and reduce the prevalence of obesity in children. Practical lessons learnt through the process included separate consideration of the law vs the implementation; enabling time for consensus; strategic alliances; and broad goal setting.
Healthy food (NYC)	Calorie Labelling in food service outlets: consider complementary strategies^56^ such as altering portion size and meal composition^57^ state and health departments ultimately succeeded after two legal challenges^49^ consider unintended consequences, for example, value for money for calories^82^ Restriction on the use of trans fats: Consider the need for health education programmes about trans fats^54 55^ including nutrition recommendations; awareness campaigns and voluntary / mandated labelling;^83^ freedom of choice argument^50^ Cap on the size of sugary beverages sold; Soda taxes have been opposed successfully in a number of US states; positive messaging better than negative campaigns;^37^ freedom of choice arguments^61 84^ can be circumvented, for example, free refills.^32^
COVID-19 Alcohol sales and transport ban (South Africa)	Raised awareness of the impact of alcohol on the community—trauma, domestic violence^22^ Unanticipated outcomes need consideration—alcohol withdrawal syndrome, including associated suicide; illegal home brewing/black market, looting, death from alcohol toxicity^29 30 44^, less informal alcohol trade=other income needed^22^ Industry opposition and lobbying need managing^22^ Consider employment/tourism implications on alcohol bans^26 74^ Given bans not sustainable, consider other measures, for example, excise taxes, minimum unit pricing, impactful health warnings, purchase limits; ban on the marketing of alcohol^51^ Other factors influencing alcohol use during COVID-19 closure of hospitality, belief that alcohol is therapeutic for COVID-19^85^
Vision Zero road safety (Sweden)	Distributing road safety responsibility beyond road users to system designers is complex^28^ and influenced by other government efforts (eg, information campaigns around car safety ratings/benchmarking) and external factors (eg, European Union directives)^39^ Facilitators^21^ include innovative thinking; institutionalised into policy with sustainable ongoing funding, demonstrated impact in Sweden; road safety education; shared commitment to goals across multiple agencies and stakeholders; and strong gov leadership, coordination and buy-in^21^
ThaiHealth (Thailand)	Strategic alliances more effective than isolated initiatives^19^ The Thai Public Broadcasting Service created a means of broadcasting health promotion efforts^19^ Four key ingredients of success—sustainable funding; strategic multisectoral approach; cutting-edge innovations; proficiency in policy advocacy and social marketing^19^ Independence from government bureaucracy enables efficient partnerships with others^19^

BMI, body mass index.

### Theory of change

Behavioural psychology underpins many of the included examples. Front of package labelling, employed in the Chile and New York City initiatives, was postulated to have dual effects—priming consumers with information that will influence their purchase and encouraging manufacturers to produce healthier products.^33–35^ Capping beverage size, known in behavioural science as manipulating ‘choice architecture’, is based on research showing that people tend to eat from bigger containers independent of their appetite or palatability of the food.^36 37^ Finally, ThaiHealth’s use of social marketing combined with strategically networked advocacy partnerships is based on the insight that awareness alone is insufficient to change behaviour—a conducive environment is also required.^19^ The remaining examples represent different approaches. The alcohol ban in South Africa related to the association of alcohol with the undermining of social distancing during the COVID-19 pandemic.^22 38^ Vision Zero represented a philosophical shift in thinking about the balance between individual responsibility and the role of system operators in contributing to road trauma and by extension, road safety^39^ (table 2A).

### Effectiveness

For all examples there was peer-reviewed research demonstrating positive effects of the interventions. Literature on food labelling showed positive effects on reformulation by manufacturers and consumer knowledge but evidence was mixed on the effect on purchasing, especially over time.^40 41^ Research demonstrated that reducing trans fats lowered hospital admissions for heart attacks and capping beverage size reduced consumption in New York.^42^ The South African alcohol bans had dramatic impacts on assaults,^43^ death rates^44^ and trauma admissions.^25^ The ban also reduced alcohol consumption, but not in problem drinkers.^45^ Vision Zero initiatives demonstrated positive impacts on road fatalities, proportional to the extent to which system changes were scaled up.^46^ ThaiHealth reported decreased smoking, alcohol consumption, alcohol-related road accidents and positive return on road safety investment^19^ (table 2B).

### Spread

Across all examples, there was widespread evidence that the strategies employed had been used in other places. Food labelling has been adopted in at least three countries other than Chile^47^; restricting of trans fats has spread to over 40 countries^42^; alcohol bans were introduced in at least 8 other countries during COVID-19^29 30^; and both Vision Zero^48^ and the ThaiHealth models have been adopted across many countries^19^ (table 2C).

### Implementation considerations

Implementation considerations broadly reflect the facilitators and barriers previously described in table 1A, B, respectively. The food labelling initiative in Chile was challenged due to lack of a legal precedent and potential impact on profits—this was countered by arguments pertaining to the obesity prevalence in children.^20^ Legal challenges^49^ and freedom of choice arguments^50^ were also successfully navigated in New York. The alcohol bans in South Africa were ultimately unsustainable due to a range of unanticipated outcomes^29^ leading to consideration of other strategies such as taxes and health warnings.^51^ As a complex, multistakeholder strategy, Vision Zero relied on a number of complementary factors such as information campaigns and a European Union directive concerning road safety management.^39^ A key facilitator for ThaiHealth was the health promotion broadcasting of the Thai Public Broadcasting Service^19^ (table 2D).

## Discussion

This study explored five successful government-led interventions to support healthier communities to identify barriers and facilitators within and across the interventions. A unique rapid review approach of a single qualitative one-on-one interview supplemented by a limited literature search was employed. Thematic analysis of the 5 interviews and 169 relevant studies across the 5 examples revealed that:

- Facilitators of success included political leadership, public education, multifaceted approaches, planning for opposition arguments and stable ongoing funding.
- Barriers included industry opposition, dealing with the complex nature of public health challenges, economic consequences that disadvantaged individuals, poor planning for flow-on effects (eg, alcohol withdrawal) and poor interagency and multisector co-ordination.
- The identified examples were generally underpinned by established behavioural science principles including priming of consumers, strategic advocacy to create change-compatible environments and reframing of the issue from an individual to a systems level.
- All examples empirically demonstrated positive impacts including changes to food manufacturing, reduced hospital admissions, reduced death rates and decreased alcohol and tobacco consumption.
- Spreading of the interventions across countries and other jurisdictions was demonstrated.

Strengths and limitations of the study warrant mention. The methodological approach was novel—rather than trying to conduct an overarching search, which would have not been feasible given the breadth of the topic, we purposefully sampled a ‘starting set’ of five examples. A similar approach was used by Webster *et al*^52^ who undertook a retrospective analysis of salt reduction programmes in four purposefully selected countries through desktop review and qualitative interviews. Their approach was more in-depth with respect to qualitative enquiry, with 8–15 people interviewed from each country; however, unlike our approach, they were not able to empirically demonstrate positive impacts of any of the initiatives because the examples were not selected on this basis.^52^ However, this example does support the viability of a combined desktop review and qualitative approach for elucidating barriers and facilitators to implementation of public health interventions.

The rationale for our minimalist review and interview approach targeting positive examples was twofold—first, to begin the process of distilling emerging ‘key ingredients’ that appear associated with success; second, to provide a framework to enable other researchers to build on this starting portfolio. Each of the mini reviews involved between 2 and 3 days of research work, including ethics, arranging the interview, conducting the Google Scholar search and writing up the example. This was designed to enable public health researchers and organisations around the world to build on the portfolio to create a progressively richer picture of government-led initiatives designed to support healthier populations. Distributing the research effort in this way carries the dual advantages of minimal unit cost and a potentially wide sampling frame. This approach also has limitations. The ‘mini-review’ approach has not been validated against more established review techniques. However, such validation is arguably more critical where point estimates of effect are required as in examples of similar interventions across different settings. Additionally, no firm conclusions on ‘what works’ were either sought or made. To sharpen the picture emerging from this exploration, more examples need to be adding to this starting portfolio using the same approach. Finally, the identified examples are predominantly ‘positive’ outliers. This means that some factors seemingly associated with success may also be present in failed initiatives, and learnings from what has not worked have not been captured. This could be addressed through sampling of further unsuccessful initiatives if the portfolio is added to by other research teams.

Commitment and leadership of government and multistakeholder support and involvement would be unsurprising to public health practitioners and researchers. Of more interest are the less-frequently reported ingredients of success, which potentially reflect more unique contexts. If the portfolio of outlier examples grows over time, it will be interesting to observe if some of these seemingly ‘unique’ factors from this starting sample of five initiatives emerge as more prominent and generalised themes. Another interesting observation is some evidence of interaction between barriers and facilitators. For example, complex multiagency approaches to public health are a ‘double-edged sword’—they are both critical to success and difficult to operationalise. The key barrier of industry opposition is somewhat predictable given that many initiatives collide with industry interests. A recently published ‘playbook’ of strategies to counter industry opposition^53^ reflects the development of counter-tactics spawned by the public health sector. Although some elements of the playbook such as linkage with social movements, creation of broad coalitions and debunking of corporate arguments are evident in the examples presented, many others such as the expansion of public health training, rigorous conflict of interest safeguards and leveraging of divergent interests or commercial tensions are less prominent. Continual learning and refinement of these strategies to optimise sustainability of public health interventions will be required to mirror the ongoing efforts of industry actors seeking to stymie public health efforts around the world.

## Conclusion

This study presents a novel approach to the daunting challenge of identifying barriers and facilitators to successful government-led interventions to support healthier populations. A rich set of themes has been elucidated from this starting portfolio of five purposefully selected examples, including the emergence of many factors operating across numerous examples. It is hoped that further examples building on this global portfolio will deepen understanding of success factors or failures over time in this critical area. Ultimately, as this portfolio builds towards a ‘saturated’ identification of facilitators and barriers of success, practitioners will be able to draw on it to enhance their efforts to address the many critical public health challenges facing citizens around the world. The author team welcome reflections on this novel portfolio approach and ideas for building on the work presented.

## Data Availability

Data are available on reasonable request. Data from this study are available on reasonable request.
