# Supplementary material for: Characteristics of successful government-led interventions to support healthier populations: a starting portfolio of positive outlier examples
Source: BMJ Glob Health. 2023 May 23;8(5):e011683. doi: 10.1136/bmjgh-2023-011683 (PMC10230917; doi:10.1136/bmjgh-2023-011683)
Supplement: Supplementary data [file bmjgh-2023-011683supp001.pdf]

Characteristics of successful government-led interventions to support healthier populations: A starting portfolio of positive outlier examples

SUPPLEMENTARY FILES

Bragge P, *et al. BMJ Global Health* 2023; 8:e011683. doi: 10.1136/bmjgh-2023-011683

Supplementary file 1: Reflexivity Statement

How does this study address local research and policy priorities?

This project explored five successful public health initiatives from around the world to elucidate an understanding of characteristics associated with success. In selecting the examples, we were conscious of the need to include those from low- and middle-income countries and therefore included examples from Thailand and South Africa which are on the current LMIC list used by the Wellcome Trust [https://wellcome.org/grant-funding/guidance/low-and-middle-income-countries]

How were local researchers involved in study design?

One person with in-depth knowledge of the public health initiative was interviewed for each of the five countries in the study. This was not necessarily a local researcher. The reason for this is that the study was exploring how a rapid snapshot of each initiative could be gathered to build a portfolio of examples over time with minimal resource cost.

How has funding been used to support the local research team?

This is not relevant to the current study because a local research team was not engaged.

How are research staff who conducted data collection acknowledged?

This is not relevant to the current study because a local research team was not engaged. The interviewees were anonymised consistent with our ethics approval for this study.

Do all members of the research partnership have access to study data?

All authors on the paper have access to data. There were no other researchers involved in this study, as the interviewees were research participants.

How was data used to develop analytical skills within the partnership?

This is not relevant to the current study because a local research team was not engaged.

How have research partners collaborated in interpreting study data?

The interviewees provided data as research participants with the interpretation undertaken by the author team who were also the research team.

How were research partners supported to develop writing skills?

The research team were all experienced academic writers with seniority ranging from a published PhD author to Professor and organisational leadership level. Therefore, development of writing skills was not a core focus of this work; although the lead author did liaise with the author team in drafting and refining the manuscript.

How will research products be shared to address local needs?

The research product of this work is in the form of this open-access academic paper. There are no other sharable research products.

How is the leadership, contribution and ownership of this work by LMIC researchers recognised within the authorship?

This is not relevant to the current study because no study authors / members of the research team are from a LMIC.

How have early career researchers across the partnership been included within the authorship team?

The author team contains an early career researcher (Alex Waddell) and a mid-career researcher (Paul Kellner)

How has gender balance been addressed within the authorship?

Five authors are male (PB, PK, RM, AN, SD) and two authors female (AW, VD)

How has the project contributed to training of LMIC researchers?

This is not relevant to the current study because no study authors / members of the research team are from a LMIC.

How has the project contributed to improvements in local infrastructure?

This project has not directly contributed to improvements in local infrastructure.

What safeguarding procedures were used to protect local study participants and researchers?

Consistent with our ethics approval, informed consent was given by all interviewees, and they were all anonymised. The topic matter was not considered sensitive or personal and therefore no safeguards for managing distress in the interviews were necessary.

Supplementary file 2: Interview Framework

Before beginning the interview, can I confirm that

- *You have read the explanatory statement*
- *You have completed the consent form [if not, obtain verbal consent and signed form]*
- *You understand I am recording this interview]*

This short interview is part of a series of case studies exploring the question:

- *What are the characteristics of successful government-led interventions to support healthier populations?*

We're adopting a very broad definition of interventions to support healthier populations, as we understand it is an inherently broad concept. Our approach to this rapid review is to gather high-level insights into a series of examples from around the world with key people such as you. The interview is focused on 'top of mind' thoughts on the key lessons from your initiative. We'll also ask you to point out any reports or other written materials that contain more details." Do you have any questions before we begin?

1. Could you please say a bit about your background and current role in relation to the intervention we're discussing today?

2. How long have you been in this role and/or other roles relating to this topic?

3. From your perspective, what was the primary aim of the intervention?

4. What level of government(s) funded the intervention? (e.g. provincial or local / state / federal)

5. Were any non-government partners involved in funding and / or delivery?
   a. If yes, briefly list

6. To date, has it worked to achieve its primary aim?
   a. If no, what are the key reasons who think it didn't work?
   b. If yes, what are the key reasons WHY it did work?
   c. (If partially successful, what were the positive and negative aspects?)

7. If you could give one piece of advice to someone doing this intervention in another part of the world, what would it be?

8. Are there any published research papers, reports or other documents that:
   a. Were used to design and develop the intervention (i.e. formative evidence); and / or
   b. Report outcomes of the intervention (i.e. summative evidence)

   If yes (to either), can you briefly supply links to these / other information (gather key authors, name of initiative, web search terms in interview – avoid having to follow up afterwards as we have tight timeframes and need to reduce admin associated with follow up)

9. Is there anything else you want to add?

Supplementary file 3: Summary of outlier examples and Google Scholar search strings

Country<br>Initiative	Google Scholar search string	N (relevant articles)
Chile<br>Front-of-package warnings on food labels containing high sugar, sodium or saturated fat[1]	"chilean|chile" "food labeling"|"nutrient warning"law|advertising|front|policy|marketing|regulation	37
New York<br>Multifaceted healthy food initiative:[2]<br>• Restriction on the use of trans fats<br>• Calorie Labelling in food service outlets<br>• Cap on the size of sugary beverages sold	"new york city|NYC" "trans fats" consumption|health|policy|obesity<br>"new york city|NYC" calorie|nutrition label|posting|count menu<br>"new york city|NYC" soda|"sugary drink" "portion sizes" reduction|health|policy|obesity	24<br>29<br>30
South Africa<br>Alcohol sales and transport ban during COVID-19[3]	"south africa" "alcohol sales" COVID-19 ban|violence|trauma|limit|policy	20
Sweden<br>Vision Zero approach to road safety[4]	"vision zero" road|traffic|safety|speed|injury|accident	13
Thailand<br>Establishment of the Thai Health Promotion Foundation - ThaiHealth[5]	"thai health promotion foundation"|"thaihealth" tobacco|smoking|alcohol|"risk factors"|"noncommunicable diseases"	16

Bragge P, *et al. BMJ Global Health* 2023; 8:e011683. doi: 10.1136/bmjgh-2023-011683

Supplementary file 4: Description of positive outlier examples

Front-of-package warnings on food labels containing high sugar, sodium or saturated fat (Chile)

One-on-one interview

Description of the initiative
The Chilean Government passed the Law of Food Labeling and Advertising in 2012. In 2016 the law went into effect. Several components aimed to change the food environment of Chile through changes to labelling and marketing of packaged foods. One part of the initiative was mandated changes to food labels of non-natural foods to display warning signs for sodium, sugar, and/or saturated fat for foods and beverages that contain nutrients in excess of specified limits. [6] Warning labels are black hexagonal boxes on the front of the package for each excess nutrient, for example “High in Sodium”. Specified limits of excess nutrients were lowered in a step-wise fashion in 2018 (phase two) and 2019 (phase three).

Context
Prior to the initiative, Chile had the highest consumption of sugar sweetened beverage in the world, and very high consumption of junk food leading to high rates of obesity and overweight in the population. [7] To combat the high rates of obesity, especially in children, the law was proposed to the Chilean senate and passed in 2012. The Ministry of health was mandated to implement the law and did so in collaboration with expert working groups.

Effectiveness of the intervention from the perspective of the interviewee and the contributing factors
The interviewee was unsure of the effect of the policy on the initial aim – to lower the prevalence of obesity – but was confident that the provision and access to unhealthy food had been lowered. They had also seen decreases in the amount of unhealthy food purchased following the implementation of the law.

“Whether it was successful in decreasing obesity, the rapid answer would be no, because we still have very high rates. But my feeling is that we have lower rates than what we would have without the implementation of the law.”

“We do see that the provision of unhealthy food has significantly decreased, and the consumption of unhealthy foods have significantly decreased. We also have information showing that marketing to children, of unhealthy foods, has decreased.”

“We also have some results from the first stage of implementation of the law, showing that the purchase of unhealthy food decrease after the implementation of the law. On the other side, on the side of the industry, we also have very consistent results, showing that the industry responded by decreasing the amounts of the nutrients that were regulated in our law. So basically what we have is a food supply that has lower content of sodium, sugar and saturated fat. So at the level of the food environment, I can say that the results were very positive, and in the direction that we anticipated with the law.”

Other Key take-outs
The interviewee felt that the complexity of food environments meant there was a need for multiple policies. These policies need to be implemented at the same time to create a multi-pronged approach to change that works in different contexts. They also felt it important to keep industry out of the conversations as they would try to undermine policy changes.

“We really need to make progress in ensuring better food environments. I would say that's very massive and complex, and that we need to consider different policies implemented at

the same time, if we really want to be successful. That means really for me, something key on the Chilean regulation, is the combination of information through labels, but also the marketing restrictions, also the protection, particularly in school environments. I think it would have been ideal to also have a tax on those unhealthy foods, and that's something that we are currently discussing here in Chile."

"I think we really need to tackle this, consider all the different angles and all the different settings in which people are exposed to unhealthy foods. That's one piece of advice. The other one is that I don't think that voluntary regulations work, that we really need to have a strong government's committed, that have the support of the academia, to really define the best policies for achieving a healthy outcome. And then we need to sit with the different actors and define how to implement this, but I think that industry or big corporates definitely cannot be part of the original discussions, or the definitions or the goals and objectives, because then things get really slow and not strong enough."

"I don't think that industry should be, or at least big corporates should be included in the discussion of defining the goals or the limits of policies, if we are really interested in promoting health."

Key differences with Chile to other jurisdictions
There are important differences in implementing policy changes in Chile compared to other jurisdictions.

"In the case of Chile, civil society didn't really play a role. Here, really the champions were a senator, a very important scientific person and the government. And in the case of the government, I think that something that really help was that in Chile, the government really has the tradition of receiving a lot of knowledge from the academia. So it's very typical that they call experts to comment or receive advice. I think that's something that I haven't seen in other places."

"And the other thing is that people who were in charge of the nutrition department in Chile, during all this long period, happened to be the same people. So it wasn't like there were a lot of changes. For different reasons, people in charge of the department remain during all these discussions. So it wasn't like we're starting again, every time that we have a change of government, it was basically a continuation. And I think that helps because sometimes you need to start from scratch once you have a new government. So I think that's something relevant to highlight, in terms of trying to find commonalities or strength from a governmental perspective, for promoting this type of actions. That's one thing."

"And the other thing is, I think it's also relevant that, in the case of Chile economists, we are also very aware of the toll that obesity and NCDs were a cause to the population. So they were already very aware of all that, those studies showing the cost effectiveness of these type of interventions. So basically, I think that the context was one in which we were already in the midst of a huge epidemic, that it was costing money to the country, and I think that economists were aware of that. So some of the discussions, I think from that economic view, were also perhaps easier to have, than in countries in which the discussion is more about preventing or avoiding having this situation."

"So I think that's different, but anyways, the economy ministry was definitely the one, with the agriculture ministry, I need to say, the two minister that were harder on approving the regulation, and the ones that resisted more the pass of the law."

Bragge P, *et al. BMJ Global Health* 2023; 8:e011683. doi: 10.1136/bmjgh-2023-011683

Information from published studies

Theory of change [how the intervention is postulated to work]
The Law of Food Labeling and Advertising was a set of policies designed to reduce the prevalence of overweight and obesity in Chile by reducing the consumption of foods and beverages high in salt, sugar, saturated fats, and energy. [8] One part of the policy package saw the addition of warning labels on foods containing high amounts of salt, sugar and saturated fats. [8] FOP labels are postulated to work by providing consumers with additional information about contents of the food or beverage thereby encouraging them to choose the healthier option. [9] It is also said to work at the industry level by encouraging manufacturers to improve their food and beverage items. [10] The World Health Organisation recommends the use of FOP as one of a number of policy level interventions to change food environments.

Effectiveness [studies empirically testing the intervention or presenting evaluation findings]
The use of front-of-pack labels on foods has gained momentum globally with labels used to depict healthier options or warn consumer of less-healthy options. [9–11] There is evidence to suggest FOP labels change food at the system level by encouraging industry through reformulation of products. [12] There is also evidence to suggest consumer’s knowledge is changed by the use of FOP. [13] However, there is little evidence for the ongoing effects of FOP on consumer behaviour. [10]

Spread [evidence that the intervention has seeded similar interventions elsewhere and / or is itself adopted from a previous intervention elsewhere]
Other countries have followed Chile’s use of FOP labels – Peru, Uruguay and Mexico. While other countries have used FOP labels for a number of years, for example, Australia that uses a star image system. Recent evaluations of the use of FOP have shown mostly favourable outcomes in the first implementation of the labels. For example, the Health Star rating in Australia was seen as practical (Pelly, 2020), while in Ecuador the use of FOP was thought to have the potential to change behaviour through informing consumer decisions (Freire, 2017). However other countries have seen some unfavourable outcomes, such as Mexico, in which consumers were mistrusting of the labels (Nieto, 2020).

Implementation considerations [barriers, facilitators, adapting to different contexts]
Villalobos Dintrans (2020) [14] provides an overview of the process of implementing The Law of Food Labeling and Advertising in Chile. They note that the implementation of the law faced several barriers – involving multiple stakeholders, political changes, and the process undertaken to define the law. The first barrier was the lack of legal precedent within Chile for FOP labelling and to make the regulation mandatory rather than optional as within other countries (for example, Australia). A literature review was conducted to establish the types of food and beverage items and the cut-off levels that should be subjected to the law. Politically, the implementation of the law faced challenges from the food and advertising industries who continued to argue against the use of FOP labels. These industry actors were concerned that the law would negatively affect profit margins. The key to arguing for the use of the law was spotlighting the need for drastic changes to affect change and reduce the prevalence of obesity in children. Five practical lessons learned through the process were identified:

1. *Broad scope of regulation: one law, three components*
2. *Separating law from its implementation: theoretical grounds versus operational details*
3. *Time for consensus: using academia as a mediator*
4. *Strategic negotiation: Reducing battle fronts and increasing allies*
5. *Goal-centered debate: Setting a common goal beyond the particular objectives of the regulation*

New York Healthy Food Initiatives

One-on-one interview

Description of the initiative

New York City has had some of the most progressive healthy food initiatives within the United States [15]. This interview explored two successful and one unsuccessful policy change to the food environment within New York city. The case report provides a comparison of policy changes across time, political leadership, and political events.
The initiatives include

1. Restriction on the use of trans fats in New York City: 2006 saw the New York City Board of Health introduced restrictions on use of trans fatty acids (TFAs) in restaurants in New York City.
2. Calorie Labelling in New York City food service outlets: In 2008 the New York City Board of Health required food chain service outlets to display calorie labelling on all menus.
3. Cap on the size of sugary beverages sold: In 2012, New York City Mayor Michael Bloomberg announced a “Sugary Drink Portion Cap Rule” to limit the size of sugary drinks sold by restaurants or retail stores to 16-ounces

Context

Food policy initiatives have long been part of the New York City fabric [15]. In 2001, Mayor Michael Bloomberg was elected mayor bringing in a number of food policy and program changes to target rising obesity, diabetes and food insecurity. Tom Freiden, a key actor in the policy and program changes, was the commissioner of the New York City Department of Health and Mental Hygiene from 2002 to 2009.

Trans Fatty Acids (TFAs) are defined as artificially made trans fats found particularly in partially hydrogenated vegetable oil (PHVO). TFAs has been shown to adversely affect the health of consumers by altering their cholesterol profile (i.e. raising low-density lipoprotein, while lowers high-density lipoprotein). Prior to the introduction of the policy, approximately 80% of New York City resident’s dietary TFAs were attributed to industrially produced PHVO.

Calorie labelling was introduced in 2008 following twenty years of research showing the link between rising sales of fast food and increased rates of obesity [16]. Consumers eat approximately 205 more calories on days they consume foods from fast food restaurants [17]. It was believed that many consumers were not aware of the higher calorie content in fast food items as this information was not readily available [16].

In 2012 NYC attempted to restrict the amount of sugary beverages sold. Sugary beverages make up the largest percentage of US adults added sugar intake (47%) [18]. These beverages are the most common beverage on sale in food, retail and other fast moving consumer goods stores [19].

The policy changes that restricted the use of TFAs in restaurants and calorie labelling came into effect swiftly and with very little back lash. There are a number of reasons for this, but according to the interviewee, the most important drivers of the success of the initiatives were involving stakeholders and preparing for arguments made by the opposition.

Involving stakeholders - the most important driver for the success of the policy was that the Health Commissioner at the time, Tom Farley, actively recruited and involved stakeholders.

“I think that's why his initiatives worked, because that's what he did. He got people personally involved. There are ways of doing successful advocacy. You get as many people on board as possible, the allies part of it was the critical part.”
Whereas the following leadership team attempting to bring in the “Sugary Drink Portion Cap Rule” did not include all the relevant stakeholders.

“You go out to the communities that you want to reach and start listening to them. Not talking at them…However they decided what they wanted to do and tried to impose it, and then got really upset because it didn't work.”

Prepare for your opposition
“They went around and told restaurant people they were doing this, demonstrated that nobody could see, taste or smell the difference [of eliminating TFAs]. Had estimates of what the difference in cost would be… So, when it was announced, there were no problems. He'd already taken care of the opposition.”

The soda tax was unsuccessful for a number of reasons, but the most crucial element for the interviewee was that the soda industry was more successful at campaigning to against the proposed legislation and were able to get large community groups on board with their message (i.e., Black and Hispanic community groups who have a long history of community support by the soda industry).
“[If you don’t include all stakeholders] you don't have a clue where the opposition is going to come from, and nobody expected the Black and Hispanic community to go with the soda company. Although if they knew the history of the soda company's involvement with Black community, they might have had a clue. But nobody expected representatives of organisations that are hit hardest by bad diets to side with the soda company. Nobody expected the City Council to side with the soda companies.”

“Nobody ever went to the Black community and discussed it with them in any way whatsoever or found out what they wanted or what their views were.”

Effectiveness of the intervention from the perspective of the interviewee and the contributing factors
According to the interviewee, the ban on TFAs in foods was successful because it was a policy that took individual’s choice away and instead focused on removing harms from their environment. Whereas the calorie labelling has proven to be ineffective because it assumes that the provision of information will change individual’s behaviour.

“The trans fat went, to the extent that you believe that trans fat is a really big problem. It's been a terrific public health measure. It's basically gone, so that worked.”

“The calorie labelling has been evaluated extensively and it basically doesn't make any difference…these labelling things, they depend on individuals reading the labels and making choices, and that's not very good public health. That's not policy. It's downstream. It's not upstream policy.”

Other Key take-outs
The underlying theory of change for the interventions are that in order to change people’s dietary habits, you have to change their environment. These policies aimed to limit people’s exposure and/or increase their awareness of the harms within their environment.

Information from published studies

Theory of change [how the intervention is postulated to work]

- **Calorie Labelling in food service outlets:** provision of information about calories will inform decision-making about food purchases [20]
- **Restriction on the use of trans fats;** adverse health effects have been demonstrated [21]
- **Cap on the size of sugary beverages sold;** changes choice architecture which means knowledge about caloric intake is less relevant; [22] people eat more from bigger containers even if not hungry and food not palatable [23]

Effectiveness [studies empirically testing the intervention or presenting evaluation findings]

- **Calorie Labelling in food service outlets:** some evidence that people who see calorie labels purchase less calories; [20,24] however awareness and impact may wane over time [25] and interpretation of meaning of messages may be difficult; [26] mixed evidence that labelling does not influence BMI [27,28] and re impact on ordering [29,30])
- **Restriction on the use of trans fats:** led to lower hospital admissions for heart attacks; [31] effective in reducing trans-fat content [32]
- **Cap on the size of sugary beverages sold:** Appeared to lower consumption when in place [33]

Spread [evidence that the intervention has seeded similar interventions elsewhere and / or is itself adopted from a previous intervention elsewhere]

- **Calorie Labelling in food service outlets:** The labelling laws spread to other states, cities and countries following their introduction in NYC [20,34] (Bernell 2010, Huang 2010)
- **Restriction on the use of trans fats:** Has been adopted in > 40 countries [31]
- **Cap on the size of sugary beverages sold:** About 40 countries have soda taxes [35]

Implementation considerations [barriers, facilitators, adapting to different contexts]

- **Calorie Labelling in food service outlets:** consider complementary strategies [36] such as altering portion size and meal composition; [37] state and health departments ultimately succeeded after two legal challenges; [38] consider unintended consequences e.g. value for money for calories [39]
- **Restriction on the use of trans fats:** Consider the need for health education programs about trans fats; [40,41] various approaches to trans fats including nutrition recommendations; awareness campaigns and voluntary / mandated labelling; [42] Argument that it reduces freedom of choice [43]
- **Cap on the size of sugary beverages sold;** Soda taxes have been opposed successfully in a number of US states due to action by soft drink companies; [23] positive messaging better than negative campaigns; [23] freedom of choice arguments; [44,45] can be circumvented (e.g. by providing free refills [46]

Alcohol sales and transport ban during COVID-19 (South Africa)

One-on-one interview

Description of the initiative
During the COVID-19 pandemic, the government of South Africa banned the sale of alcohol as a non-essential product. The first ban coincided with the first lockdown order of 15 days and was extended with each subsequent lockdown. Between each lockdown, alcohol sales resumed.

Context
South Africa has some of the highest rates of alcohol related morbidity and mortality in the world, ranking 6th in the world for average alcohol consumption per day by the population [47]. The resources needed to treat alcohol related trauma are the same that are needed to treat COVID-19 in hospitals (e.g. ventilators). The restriction on the sale of alcohol was put in place to protect the health care systems and save resources for COVID-19 related treatment.

"We are the sixth highest nation in terms of the amount of alcohol consumed per drinker per day. It causes a lot of harm."

In order to continue the ban on alcohol sales, the Medical Research Council established a team of alcohol epidemiologists and trauma doctors to model the effects of the restrictions on the healthcare system. These results were translated into relative terms (i.e. trauma admissions saved, ventilators, and hospital beds). The economic modelling showed that a ban on alcohol sales would result in 48,000 admissions to trauma units saved. Armed with this information, the South African government implemented an ongoing ban on the sale of alcohol in line with COVID-19 lockdowns.

"We could show that alcohol had huge impact on infectious diseases, non-infectious disease is non-infectious diseases and trauma mental health, and presented that to this parliamentary grouping. And they felt quite shocked."
"We had four liquor bans in South Africa, comprising I think a total of 164 days over an 18-month period."

Effectiveness of the intervention from the perspective of the interviewee and the contributing factors
From the perspective of the interviewee, the intervention was extremely successful while in place.

"At one stage, this one paper shows how it was saving about 72 lives a day…But it was on average about 45 lives a day, because of the alcohol bans"
"There were two papers on trauma, and there were two papers on death data [showing the success of the initiative."

The initiative was said to work because the researchers were able to work with government during a window of opportunity to use long-standing research on the effects of alcohol in South Africa to implement policy changes.

"When there's a situation when there are multiple options, and the research suggests a way forward and helps them. Policymakers have very short time focuses."

In addition, researchers were ready with the information that the government needed and were willing to put themselves out in the public sphere to defend the research.

“You need people who have the information that's available at the time you need it. So, we've been working for 25 plus years, and vaguely sometimes get listened to, often not. But in a way, looking at critically the fact that as a country we had, it wasn't just me. We had people who were already in waiting who'd got research, who'd got contacts, who weren't afraid to take risks. You've got to put yourself out there.”

“So you got to have people who are willing to say, "This public health crisis is big enough. We've got to make bold statements, and we've got to do modelling even with incomplete data."

They also spoke to the importance of using multidisciplinary teams to collaborate on the problem.

“You have to have multidisciplinary teams and drawing people as you need them. And I think building the coalition, I certainly didn't want to stick my head out there, so I brought in other experts and civil society people and worked very hard with the media to defend the policy. And there were multiple engagements. I was contacted by cabinet ministers often, and I would speak on the phone and send them research to back up what I was trying to say. So it gave me an opportunity to feed in things I've been trying to get people to listen to for years. So the crisis brought the government and the researchers together in a way that doesn't always happen. You have to have a pandemic or something like that.”

Other Key take-outs

The initiative was unable to be used over the long term. Prior to the end of the fourth ban the Medical Research Council lobbied the government to establish a basket of less intrusive policies aimed at reducing the harms of alcohol sales. Unfortunately, none of these initiatives were implemented by the South African Government

“We thought we should have a basket of more realistic measures, which would be more things that we could sustain in the long term.”

Following the end of the ban on alcohol sales, there was an increase in alcohol related trauma, including the death of 21 underage people from methanol poisoning. However, there continues to be no decisive action from the South African government

“So, things returned quite quickly to normal. We've had a couple of incidents recently, which have really shown that actually... And the government's saying, "We need to do something more about alcohol again...But it seems that the lessons were not really learnt."

The interviewee developed a 10-point plan for combating alcohol related morbidity and mortality for UNHCR. [48]

1. Agree on <u>clear plan</u> with clear objectives, timelines, delivery mechanisms & outcomes
2. Ensure appropriate level of <u>excise taxes</u>
3. Adequate controls of <u>alcohol marketing</u> & promotions, including internet marketing
4. Limit availability of <u>products with higher risk</u> for promoting heavy drinking, e.g. larger container sizes, lower priced products (through minimum unit pricing)
5. Improve <u>drink driving countermeasures</u> (lower maximum BAC limits, enforcement & timely consequences)
6. More controls on <u>alcohol availability</u> (sales through unregistered outlets, outlets in high density or high-risk areas, home deliveries)
7. Get tough on <u>illicit/unrecorded</u> alcohol through tracking & tracing system
8. Increase availability of <u>treatment options</u> for person struggling with heavy drinking/alcohol dependence

9. Establish mechanisms to monitor & evaluate implementation of plan starting with sentinel surveillance of alcohol-related trauma
10. Ensure mechanism for translating plan into action: leadership, accountability, financing, balancing competing interests within government, manage liquor industry conflicts of interest, community mobilisation

Information from published studies

Theory of change [how the intervention is postulated to work]

- Alcohol use associated with undermining of social distancing and compromising immune response

Effectiveness [studies empirically testing the intervention or presenting evaluation findings]

- Led to 80% decrease in rapes and aggravated assaults [49] and having of the unnatural death rate from 800 – 1000 to 400 / week [50]
- Reduction on trauma admissions [51,52] greatest for violence-related trauma [53]
- Reduces consumption, but not in problem drinkers [54]

Spread [evidence that the intervention has seeded similar interventions elsewhere and / or is itself adopted from a previous intervention elsewhere]

- Other countries that instituted pandemic-associated alcohol restrictions included India, Nepal, Slovenia, India and Thailand, [55] Georgia, Greenland and Russia [56]

Implementation considerations [barriers, facilitators, adapting to different contexts]

- Raised awareness of the impact of alcohol on the community – trauma, domestic violence decreased during the ban represents a window of opportunity [57]
- Unanticipated outcomes need consideration – alcohol withdrawal syndrome, including associated suicide; illegal home brewing / black market, looting, death from alcohol toxicity [50,55,56] – this led to France reversing their alcohol ban [56]
- Industry opposition and lobbying need managing [57]
- Cascade effects need attention – e.g. about 50,000 engage in the informal alcohol trade and need alternative sources of income [57]
- Lifting of bans should be phased to prevent return to binge drinking [57]
- Consider employment / tourism implications on alcohol bans [53,58]
- Given bans are not sustainable, consider other measures e.g. raising excise taxes, minimum unit pricing, impactful health warnings, upper limits on amounts of alcohol that may be delivered / purchased, ban on the marketing of alcohol except at points of sale [59]
- Consider other factors influencing alcohol use in COVID context including closure of hospitality venues and misunderstandings about alcohol as a therapeutic COVID intervention [60]

Vision Zero approach to road safety

One-on-one interview

Description of the initiative
Vision Zero is "an ethical approach to road safety that calls for greater participation of multiple stakeholders such as road users, system designers, vehicle industries, public health professionals, and local governments" (Mendoza 2017 p. 104)[61]. Examples of Vision Zero interventions include crash barriers; using government fleet buying power to mandate safer cars; and reducing speed limits to 30 km / h in urban areas with high cyclist or pedestrian traffic.

Context
The initiative was instigated by someone with deep inside knowledge of the policy environment. There was immediate acceptance by the Swedish Minister of Infrastructure when it was first proposed in 1995, and it was legislated in 1997. In part this is because of the **power of the idea and widespread political support**:

> *"it becomes impossible to say no, because you would be seen as cold-hearted … we knew the minister came from the occupational health and safety area. She picked up that very quickly and made it her own saying, "Road traffic should be as the workplace. Everyone should be expected to come home alive after a workday or in road traffic."*
>
> *"The Swedish Parliament voted for VZ in October 1997, and all parties were in favor. One party had a minor alteration of the proposal, but in essence all were positive. No political party or any Minister of Transport has ever openly questioned that decision since"* (Tingvall 2022 p. 9)[62].

The importance of a **mindset that shifts from personal responsibility to system responsibility and the political will to make this happen** was emphasised as a key barrier:

> *There are some countries … that are not that poor, but they haven't done the slightest to reduce the road toll … The government has left sort of everything that deals with safety to the citizen … And they don't even care for the government fleet of vehicles … they have a horrendous road toll. I mean, you're talking 20 times worse or something like that than our country.*
>
> *We had so many people from so many countries coming to us in Sweden … And you said, "Give me some paint, and give me a way to dig holes, and we can completely change." To build a speed hump outside schools, where you got very high speeds and things like that is so simple … we can sort of make people understand by perception what sort of it is, you can do great things. Normally it's actually the police who is responsible for traffic safety in a country … it's the lone perpetrator that is there what they're talking about. It's that mindset. And it's very, very hard to sort of talk about change in that mindset.*

Another potential success factor was that **not all the interventions associated with Vision Zero were costly**:

> *"I even personally was sort of against talking about that you need so much more resources. We need commitment. We need decisions taken. We need to apply the scientific-based approach to everything that we're doing … We have to reduce speed to 30 kilometres per hour in urban areas where you mix pedestrians and bicyclists and cars … That doesn't cost anything more than political will and the courage to present what this is all about, the knowledge that you don't reduce mobility by doing that"*

A key success factor is that **the 'vision' needs to be backed up by interventions**

> *Vision Zero is a way to get the interventions done, and the mindset changed, and the responsibility transferred over to other stakeholders of the community. Sweden has had sort of a successful … But I mean, it's based on that you actually do things … … If you improve the car fleet, you replace maybe 5%, 6% of the car fleet every year. In one year you can't do very much. On the other hand, if you do it for many years … you will get quite fantastic results*

Effectiveness of the intervention from the perspective of the interviewee and the contributing factors

Sweden's road toll has dropped from about 600 per year to 200 since Vision Zero was instigated.

Other Key take-outs [combination of Q7 and other key themes / impactful quotes]

> *if you have poor safety, parents won't send the kids to school by walking. If children don't walk to school, soon or later, that will hit their health … That is where the road safety community is going now, slowly but still understanding there are other benefits from safety*

> *public procurement is 15%, 20% of the economy of a country. Public procurement is a really fantastic lever to get things done out there, because everyone will need to adjust to it.*

> *If you build in sustainability, both carbon footprint, safety footprint and so on, and a few things around that, or health or whatever it could be, sure, of course it has a major impact on the community … Road safety needs to get out there in the community with other needs of the community, understand that we are part of something bigger called sustainability*

> *you look at value chains then, or supply chains, that probably kills some 500,000 people in road traffic every year, roughly … And measuring the safety footprint of big corporations, that's what's sort of going on now, those kinds of things, and includes procurement and public procurement. And then you're talking about not only in your country, but sort of what happens with the supply chains for BHP or whatever somewhere is going to be counted and going to be a part of sustainability reporting. And of course, a company or corporation needs to work on that*

Information from published studies

Theory of change [how the intervention is postulated to work]

Vision Zero is based on the ideas that responsibility for road safety is not limited to the actions of road users; it is also a responsibility of system operators: *"tradition and road traffic rules for the road users have been used as an excuse for not undertaking necessary system changes and modifications" (p. 2) … "It is human to make mistakes, and we must design for the human as we are, not the perfect human that in reality does not exist" … (p. 4).* System modifications, for example airbags and road safety barriers, are designed based on strategies to *"control, harness, reduce, cushion, or redirect harmful kinetic energy"*, which was described as a key contributor to road traffic injury and death as far back as 1970.[63]

Ways of holding road users accountable through road laws such as speed and alcohol limits are well understood. The theory of change for system designers is similar, but based on formal regulations and road safety standards, for example mandating airbags in car manufacturing.[64]

Effectiveness [studies empirically testing the intervention or presenting evaluation findings]

It is difficult to establish the effect of system-level interventions such as Vision Zero given their system-wide nature, but there is evidence that system changes have positive impacts. An early example from Sweden was the implementation of "2 + 1" roads, in which a standard 2-lane road is converted to three lanes to create an overtaking lane which alternates every few kilometres, with the two directions separated by a physical barrier.[65] This was reported to reduce risk of fatality by 80%.[62] Lowering speed limits has been shown to reduce fatalities.[66][67] At a whole-of country level, Johansson et al. (2009) reported a reduction in road deaths per 100,000 people from 6 to 4.7 in the decade following implementation of Vision Zero. The authors also report that large-scale attempts to implement design principles have been made, fatalities can be reduced by up to 90% compared to 2 – 3% reduction in areas where no such improvements have been made.[66]

However, the global road death picture is not as positive. The Decade of Action for Road Safety 2011 – 2020 aimed to stabilise, then reduce the global number of road fatalities. Although global road deaths are below the 1.9 million in a 'no action' scenario, these aims were not met. The current target, part of the UN Sustainable Development Goals, is to halve global deaths and serious injuries from road traffic accidents.[68]

Spread [evidence that the intervention has seeded similar interventions elsewhere and / or is itself adopted from a previous intervention elsewhere]

The Vision Zero approach has been adopted across many countries including Norway, Australia, New Zealand, Poland UK, Germany and the United States and India. However, implementation is in various stages and implementation challenges have been reported, for example political commitment and funding.[69][61]

Implementation considerations [barriers, facilitators, adapting to different contexts]

A key challenge in establishing effectiveness is the complexity of distributing road safety responsibility beyond road users to system designers – for example administrators, car manufacturers and transport providers. This requires reframing of the problem beyond individual behaviour change to a system-wide issue.[70]

The process of operationalising this – which involves legislation, regulation, government and private sector investment - involves navigating complexity (for example, legal frameworks are designed around liabilities of individual road users) and conflicts of interest (benefits are to all; costs borne by administrators). This is influenced by other government efforts (e.g. information campaigns around car safety ratings / benchmarking) and external factors (e.g. EU directives).[64]

Ahmadi (2020) outlines a number of facilitating factors which support Vision Zero as an exemplar practice:

- Innovative way of thinking;
- Institutionalised into policy with sustainable ongoing funding and demonstrated impact in Sweden;
- Educates the public regarding road safety behaviours;
- Advocates and implements engineering solutions underpinned by legislation;
- Shared commitment to goals across multiple agencies and stakeholders; and
- Strong leadership and coordination across all levels of government, with a high level of buy-in from stakeholders.[71]

Establishment of the Thai Health Promotion Foundation – ThaiHealth

Description of the initiative [short, including info from Q 3 – 5 of the interview framework]:

The Thai Health Promotion Foundation (ThaiHealth) is an autonomous government body that seeks to address the multisectoral aspects of noncommunicable disease prevention in Thailand.

***Context [reflect on political climate, country-level issues and other system-level factors that either drove or influenced the initiative e.g. COVID, rising obesity rates, too many deaths on the road]*:**

ThaiHealth was initiated by senior public health leaders who were seeking to build on the Ottawa Charter and, in part, inspired by the innovative financing approach that established the Victorian Health Promotion Foundation, VicHealth. Similar to VicHealth's tobacco tax, ThaiHealth's uses a 2% excise tax on tobacco and alcohol to fund health promotion interventions. The public health leaders' efforts ultimately led to parliament establishing ThaiHealth by enacting the Thai Health Promotion Foundation act in 2001.

It **focuses and capitalises on synergies between scientific evidence, political involvement, and social movements** to improve health lifestyles and practices, as well as improve healthy environments. This is sometimes referred to as "**moving the mountain**" because the three prongs of the approach are depicted within a triangle resembling a mountain:

> *"… if we have combined these three powers, you can move any difficulties because the politic is very sensitive to topic scrutiny."*

The initiative is strengthened not only by a legislative basis but also the **senior level political engagement** at its centre:

> *"[ThaiHealth] is governed by a board chaired by Prime Minister…and it is a multi-sectoral multi-stakeholder governing board [which includes] …national government…local parliament representatives as well…"*

Involving **civil society and social movements ensures accountability and transparency**:

> *"…also [there is] a subcommittee with representation from CSOs…When you involve a civil society representative in the policy process, they are very transparent, and they are held accountable. So that is the gist of how we manage in many fronts…"*

ThaiHealth was also **rooted in scientific evidence**. Analysis of epidemiological data were used to identify key focus areas, including:

> *"…tobacco and alcohol, unhealthy diet, safe environment, [and] work injury prevention."*

Using tax revenue to health promotion activities, as opposed to healthcare services, is seen to be wise because it ultimately seeks to resolve the issues of concern:

> *"In some countries they use earmark sin tax to fund health services like free health. In [another country], they use tobacco tax to fund health services. Which is a never-ending hollow bucket, but Thai health never fund this health services, but they fund active health promotion, empower people [towards] healthy [lifestyles] and look at the critical pinch point. Tobacco is our pinch point, alcohol is pinch point, drunk driving is our pinch point and healthy diet is a pinch point."*

Effectiveness of the intervention from the perspective of the interviewee and the contributing factors [distil from Q6 of the interview framework]

ThaiHealth's explicit focus on prevention and primary prevention in relation to structural and social determinants of health was seen as the core reason for the institution being effective.

> *"we address…key structural determinants and social determinants of health very effectively. If we go and treat the proximal determinants, we never ending story and expenditure so high compared to prevention. If we can reduce alcohol consumption by X% or reduce sugar consumption by Y%, we can reduce the number, how many cases of obesity we can prevent?* It is so cost effective to invest on prevention and primary prevention and address the *social determinants of* health."

Information from published studies

Theory of change [how the intervention is postulated to work]

- "The 'Triangle that Moves the Mountain' is a conceptualized strategy initiated as a social tool for solving difficult social problems, by simultaneously strengthening capacity in three interrelated sectors: (1) creation of knowledge; (2) social movement; and (3) political involvement." [72]
- The model that they apply involves building "capacities of communities, government and non- government organizations, public interest organizations, state enterprises and agencies to plan, develop and conduct their own health promotion programmes." [73]
- Social marketing is combined with strategically networked advocacy partnerships to promote both awareness-raising and behaviour change – based on the realisation that awareness alone is not sufficient for behaviour change; a conducive environment is also required [74]
- Using a 2% excise tax on alcohol and tobacco to undertake health promotion work [5,75,76]
- Senior-level involvement in leadership – the ThaiHealth Board chair is the Prime Minister of Thailand [75]

Effectiveness [studies empirically testing the intervention or presenting evaluation findings]

- Decrease in smoking prevalence from 25.47% to 19.94% [74]
- 13% decrease in alcohol consumption [74]
- Alcohol free Buddhist lent period led to 20% reduction in road accidents from drunk driving [74]
    - a related social marketing campaign found that using a strategy that factors in exposure marketing supportive of alcohol consumption as well as a ThaiHealth campaign to reduce alcohol consumption was effective – those who saw both standard alcohol ads and the social marketing campaign were had the highest probability of reducing their alcohol consumption [77]
- Road safety return on investment 130.2 baht for each 1 baht invested [74]
- Decline in death rate from vehicle accidents from 22.9 per 100 000 in 2003 to 16.82 per 100 000 in 2010 [75]
- Social return on Investment figures from 2014 study [78]
    - Food and Nutrition programs – 13.49 (13.49 baht value for 1 baht spent)
    - Programs for disabled persons – 1.18
    - Elderly programs – 2.95

Bragge P, *et al. BMJ Global Health* 2023; 8:e011683. doi: 10.1136/bmjgh-2023-011683

- Programs for children and youth – 6.87 [78]
- ThaiHealth's work helped support a 4.6 fold increase in Thai publishing about tobacco-related research (from 74 papers in the first 10 years analysed to 376 in the second 10 years analysed) [79]
- A study about interventions to change collective social norms in Thailand observed the "availability of ThaiHealth's financial support serving as catalytic funding for the intervention implementations and research funding for the generation of the indirect evidence in the longer term." [80]
- "The percentage of the adult population doing at least 150 minutes of moderate-intensity or 75 minutes high-intensity aerobic exercise per week, increased from 66.3% in 2012 to 72.9% in 2017" [81]

Spread [evidence that the intervention has seeded similar interventions elsewhere and / or is itself adopted from a previous intervention elsewhere]

- ThaiHealth has supported establishment of similar organisations in Malaysia, South Korea, Mongolia and Tonga [74]
- There is an International Network of Health Promotion Foundations further reflecting global spread – Austria, Taiwan as well as the above countries [74]
- Played a key role in establishing the Center for Alcohol Studies of Thailand [82]
- Tobacco control researchers that started some of their work through ThaiHealth continue their work through a wide range of international funding support and collaboration with organisations like the Rockefeller Foundation, Bloomberg Philanthropies, and Johns Hopkins University [79]
- ThaiHealth and affiliated researchers developed tools for engaging in cross-national comparison, for instance an measure on Harm to Others from Drinking [83,84]

Implementation considerations [barriers, facilitators, adapting to different contexts]

Facilitators

- Strategic alliances have been found to be more effective than isolated initiatives [74]
- The Thai Public Broadcasting Service (Thai PBS) was established in response to difficulties finding broadcast channels from early health promotion efforts [74]
- Key ingredients of success identified:
  - Sustainable funding [74,75]
  - Strategic multi-sectoral approach [74]
  - Cutting-edge innovations e.g. alcohol-free Buddhist Lent [74]
  - Proficiency in policy advocacy and social marketing [74]
  - Flexible approach [85]
- Independence from gov bureaucracy enables efficient partnerships with others [74]
- Well-timed, well-targeted, well-communicated evidence being shared with the public has supported their objectives in multiple cases [72]

Barriers

- Broad shifts in who consumes alcohol towards previously abstinent groups like youth and women [86]

Adapting to different contexts

- ThaiHealth transformed the health promotion landscape by consolidating a range of actors' efforts around tobacco control [87]
- ThaiHealth learned from their engagement on e-cigarettes and related products that it's important to continually engage the public and policymakers around how tobacco companies might develop alternative markets for their products [88]

References

1 Taillie LS, Bercholz M, Popkin B, *et al.* Changes in food purchases after the Chilean policies on food labelling, marketing, and sales in schools: a before and after study. *Lancet Planet Heal* 2021;**5**:e526–33. doi:10.1016/S2542-5196(21)00172-8

2 Farley T. *Saving Gotham : a billionaire mayor, activist doctors, and the fight for eight million lives*. New York: : Norton & Company 2015.

3 Theron M, Swart R, Londani M, *et al.* Did COVID-19-Related Alcohol Sales Restrictions Reduce Alcohol Consumption? Findings from a National Online Survey in South Africa. *Int J Environ Res Public Health* 2022;**19**:2422. doi:10.3390/ijerph19042422

4 Edvardsson Björnberg K, Belin M, Hansson S., *et al. The Vision Zero Handbook*. Springer, Cham 2022.

5 Pongutta S, Suphanchaimat R, Patcharanarumol W, *et al.* Lessons from the Thai Health Promotion Foundation. *Bull World Health Organ* 2019;**97**:213–20. doi:10.2471/BLT.18.220277

6 Kanter R, Reyes M, Vandevijvere S, *et al.* Anticipatory effects of the implementation of the Chilean Law of Food Labeling and Advertising on food and beverage product reformulation. *Obes Rev* 2019;**20**:129–40. doi:10.1111/obr.12870

7 Caro JC, Ng SW, Taillie LS, *et al.* Designing a tax to discourage unhealthy food and beverage purchases: The case of Chile. *Food Policy* 2017;**71**:86–100. doi:10.1016/j.foodpol.2017.08.001

8 Zancheta Ricardo C, Corvalan C, Tallie L, *et al.* Non-nutritive sweeteners in the food supply: changes after the first phase of the Chilean Food Law. *Eur J Public Health* 2020;**30**. doi:10.1093/eurpub/ckaa165.576

9 Nestle M. Public health implications of front-of-package labels. Am. J. Public Health. 2018. doi:10.2105/AJPH.2017.304285

10 Croker H, Packer J, Russell SJ, *et al.* Front of pack nutritional labelling schemes: a systematic review and meta-analysis of recent evidence relating to objectively measured consumption and purchasing. *J Hum Nutr Diet Off J Br Diet Assoc* 2020;**33**:518–37. doi:10.1111/jhn.12758

11 Springmann M, Clark M, Mason-D'Croz D, *et al.* Options for keeping the food system within environmental limits. *Nature* Published Online First: 2018. doi:10.1038/s41586-018-0594-0

12 Vyth EL, Steenhuis IHM, Roodenburg AJC, *et al.* Front-of-pack nutrition label stimulates healthier product development: A quantitative analysis. *Int J Behav Nutr Phys Act* Published Online First: 2010. doi:10.1186/1479-5868-7-65

13 Hersey JC, Wohlgenant KC, Arsenault JE, *et al.* Effects of front-of-package and shelf nutrition labeling systems on consumers. *Nutr Rev* Published Online First: 2013. doi:10.1111/nure.12000

14 Villalobos Dintrans P, Rodriguez L, Clingham-David J, *et al.* Implementing a Food Labeling and Marketing Law in Chile. *Heal Syst Reform* 2020;**6**:1–8. doi:10.1080/23288604.2020.1753159

15 Freudenberg N, Cohen N, Poppendieck J, *et al.* Food policy in New York City since 2008: Lessons for the next decade. *CUNY Urban Food Policy Inst Guid to Food Gov New York City* Published Online First: 2018.http://www.cunyurbanfoodpolicy.org/news/2018/2/16/food-policy-in-new-york-city-since-2008-lessons-for-the-next-decade

16 Dumanovsky T, Huang CY, Bassett MT, *et al.* Consumer awareness of fast-food calorie information in New York City after implementation of a menu labeling regulation. *Am J Public Health* 2010;**100**:2520–5.doi:10.2105/AJPH.2010.191908) (accessed 3 Oct 2022).

17 Paeratakul S, Ferdinand DP, Champagne CM, *et al.* Fast-food consumption among US adults and children: Dietary and nutrient intake profile. *J Am Diet Assoc* 2003;**103**:1332–8. doi:10.1016/S0002-8223(03)01086-1

18 McGuire S. Scientific Report of the 2015 Dietary Guidelines Advisory Committee. Washington, DC: US Departments of Agriculture and Health and Human Services, 2015. *Adv Nutr* 2016;**7**:202–4. doi:10.3945/AN.115.011684

19 Adjoian T, Dannefer R, Sacks R, *et al.* Comparing sugary drinks in the food retail environment in six NYC neighborhoods. *J Community Health* 2014;**39**:327–35. doi:10.1007/S10900-013-9765-Y/FIGURES/2

20 Bernell B. The history and impact of the new york city menu labeling law. Food Drug Law J. 2010;**65**:839–72.https://heinonline.org/hol-cgi-bin/get_pdf.cgi?handle=hein.journals/foodlj65&section=41 (accessed 3 Oct 2022).

21 Willett W. The Case for Banning Trans Fats. *Sci Am* 2014;**310**:13–13. doi:10.1038/scientificamerican0314-13

22 Cohen DA, Story M. Mitigating the health risks of dining out: The need for standardized portion sizes in restaurants. Am. J. Public Health. 2014. doi:10.2105/AJPH.2013.301692

23 Giolitto SU. *Normative portions: alignment of descriptive and injunctive norms for portion size reduction*. 2015;**13**.

24 Bollinger B, Leslie P, Sorensen A. Calorie posting in chain restaurants. *Am Econ J Econ Policy* 2011;**3**:91–128. doi:10.1257/pol.3.1.91

25 Cantor J, Torres A, Abrams C, *et al.* Five years later: Awareness Of New York City's calorie labels declined, with no changes in calories purchased. *Health Aff* 2015;**34**:1893–900. doi:10.1377/HLTHAFF.2015.0623

26 Cohn EG, Larson EL, Araujo C, *et al.* Calorie postings in chain restaurants in a low-income urban neighborhood: Measuring practical utility and policy compliance. *J Urban Heal* 2012;**89**:587–97. doi:10.1007/S11524-012-9671-0

27 Drichoutis AC, Nayga, Jr. RM, Lazaridis P. Can Nutritional Label Use Influence Body Weight Outcomes? *Kyklos* 2009;**62**:500–25. doi:10.1111/j.1467-6435.2009.00448.x

28 Restrepo BJ. Calorie Labeling in Chain Restaurants and Body Weight: Evidence from New York. *Heal Econ (United Kingdom)* 2017;**26**:1191–209. doi:10.1002/hec.3389

29 Roberto CA, Larsen PD, Agnew H, *et al.* Evaluating the impact of menu labeling on food choices and intake. *Am J Public Health* 2010;**100**:312–8. doi:10.2105/AJPH.2009.160226

30 Long MW, Tobias DK, Cradock AL, *et al.* Systematic review and meta-analysis of the impact of restaurant menu calorie labeling. *Am J Public Health* 2015;**105**:e11–24. doi:10.2105/AJPH.2015.302570

31 Ghebreyesus TA, Frieden TR. REPLACE: a roadmap to make the world trans fat free by 2023. *Lancet* 2018;**391**:1978–80. doi:10.1016/S0140-6736(18)31083-3

32 Rahkovsky I, Martinez S, Kuchler F. New food choices free of trans fats better align U.S. diets with health recommendations. In: *Americans and Food Choices: Select Research on Time and Diet*. 2012. 114–52.https://ageconsearch.umn.edu/record/291933/ (accessed 3 Oct 2022).

33 Kennelly M. Downsizing : New York City's Attempt to Shrink Portion Sizes NYC ' s sugary drink portion cap rule. In: *Buena Salud Americas Conference 2015*. 2015.

34 Huang C, Dumanovsky T. A brief overview of New York City's calorie labeling

regulation and evaluation. *Nutr Today* 2010;**45**:226–8. doi:10.1097/NT.0b013e3181f1d715
35 Beebe J. Should There Be a Soda Tax? *Rice Univ Bak Inst Public Policy* 2019.
36 Gordon C, Hayes R. Counting Calories: Resident Perspectives on Calorie Labeling in New York City. *J Nutr Educ Behav* 2012;**44**:454–8. doi:10.1016/j.jneb.2012.01.004
37 Elbel B, Kersh R, Brescoll VL, *et al.* Calorie labeling and food choices: A first look at the effects on low-income people in New York City: Calorie information on menus appears to increase awareness of calorie content, but not necessarily the number of calories people purchase. *Health Aff* 2009;**28**:w1110–21. doi:10.1377/hlthaff.28.6.w1110
38 Farley TA, Caffarelli A, Bassett MT, *et al.* New York City's fight over calorie labeling: A two-year struggle ultimately proves that innovation in food regulation is entirely possible at the local level. *Health Aff* 2009;**28**:w1098–109. doi:10.1377/hlthaff.28.6.w1098
39 Loewenstein G. Confronting reality: pitfalls of calorie posting. *Am J Clin Nutr* 2011;**93**:679–80. doi:10.3945/ajcn.111.012658
40 Kamel S, Al Otaibi H. Trans-fats declaration, awareness and consumption in Saudi Arabia. *Curr Res Nutr Food Sci* 2018;**6**:748–56. doi:10.12944/CRNFSJ.6.3.17
41 Remig V, Franklin B, Margolis S, *et al.* Trans Fats in America: A Review of Their Use, Consumption, Health Implications, and Regulation. *J Am Diet Assoc* 2010;**110**:585–92. doi:10.1016/j.jada.2009.12.024
42 L'Abbé MR, Stender S, Skeaff CM, *et al.* Approaches to removing trans fats from the food supply in industrialized and developing countries. *Eur J Clin Nutr* 2009;**63**:S50–67. doi:10.1038/ejcn.2009.14
43 Resnik D. Trans fat bans and human freedom. *Am J Bioeth* 2010;**10**:27–32. doi:10.1080/15265160903585636
44 Gostin L. The New York City "Soda Wars": Public Health vs Paternalism. *JAMA Forum Arch* 2013;**A2**. doi:10.1001/JAMAHEALTHFORUM.2013.0016
45 Gostin LO, Reeve BH, Ashe M. The historic role of boards of health in local innovation. *JAMA* 2014;**312**:1511–2. doi:10.1001/jama.2014.12498
46 John LK, Donnelly GE, Roberto CA. Psychologically Informed Implementations of Sugary-Drink Portion Limits. *Psychol Sci* 2017;**28**:620–9. doi:10.1177/0956797617692041
47 WHO. Global status report on alcohol and health 2018. 2019.
48 Parry C, Gray G, Maker A, *et al.* Charting a healthier way forward for alcohol in SA, now and into the future. South African Med. Res. Counc. 2020.
49 Lange S, Probst C, Rehm J. Coronavirus disease 2019 crisis and intentional injuries: now is not the time to erode alcohol control policies. *Can J Public Heal* 2020;**111**:466–8. doi:10.17269/s41997-020-00391-6
50 Reuter H, Jenkins LS, De Jong M, *et al.* Prohibiting alcohol sales during the coronavirus disease 2019 pandemic has positive effects on health services in South Africa. *African J Prim Heal Care Fam Med* 2020;**12**. doi:10.4102/phcfm.v12i1.2528
51 Navsaria PH, Nicol AJ, Parry CDH, *et al.* The effect of lockdown on intentional and nonintentional injury during the COVID-19 pandemic in Cape Town, South Africa: A preliminary report. *South African Med J* 2020;**111**:110. doi:10.7196/SAMJ.2021.v111i2.15318
52 van Hoving DJ, van Koningsbruggen C, de Man M, *et al.* Temporal changes in trauma according to alcohol sale restrictions during the South African national COVID-19 lockdown. *African J Emerg Med* 2021;**11**:477–82. doi:10.1016/j.afjem.2021.08.001
53 Navsaria P, Nicol A, Parry C, *et al.* The effect of lockdown on international and non-

intentional injury during the COVID-19 pandemic in Cape Town, South Africa: A preliminary report. *South African J Econ* 2020;**14**.

54 Myers B, Carney T, Rooney J, *et al.* Alcohol and Tobacco Use in a Tuberculosis Treatment Cohort during South Africa's COVID-19 Sales Bans: A Case Series. *Int J Environ Res Public Health* 2021;**18**:5449. doi:10.3390/ijerph18105449

55 Calvey T, Scheibein F, Saad NA, *et al.* The Changing Landscape of Alcohol Use and Alcohol Use Disorder During the COVID-19 Pandemic - Perspectives of Early Career Professionals in 16 Countries. *J Addict Med* 2020;**14**:e284–6. doi:10.1097/ADM.0000000000000735

56 Neufeld M, Lachenmeier DW, Ferreira-Borges C, *et al.* Is Alcohol an "Essential Good" During COVID-19? Yes, but Only as a Disinfectant! *Alcohol Clin Exp Res* 2020;**44**:1906–9. doi:10.1111/acer.14417

57 Matzopoulos R, Walls H, Cook S, *et al.* South Africa's COVID-19 alcohol sales ban: The potential for better policy-making. Int. J. Heal. Policy Manag. 2020;**9**:486–7. doi:10.34172/ijhpm.2020.93

58 Rogerson CM, Rogerson JM. COVID-19 Tourism impacts in South Africa: Government and Industry Responses. *Geoj Tour Geosites* 2020;**31**:1083–91. doi:10.30892/gtg.31321-544

59 Parry CDH. A timely piece that resonates with the South African experience: Commentary on Stockwell et al . *Drug Alcohol Rev* 2021;**40**:8–9. doi:10.1111/dar.13159

60 Ramalho R, Adiukwu F, Gashi Bytyçi D, *et al.* Alcohol and Tobacco Use During the COVID-19 Pandemic. A Call for Local Actions for Global Impact. *Front Psychiatry* 2021;**12**. doi:10.3389/fpsyt.2021.634254

61 Mendoza AE, Wybourn CA, Mendoza MA, *et al.* The Worldwide Approach to Vision Zero: Implementing Road Safety Strategies to Eliminate Traffic-Related Fatalities. *Curr Trauma Reports* 2017;**3**:104–10. doi:10.1007/s40719-017-0085-z

62 Tingvall C. Vision Zero: How It All Started. In: *The Vision Zero Handbook*. Cham: : Springer International Publishing 2022. 1–22. doi:10.1007/978-3-030-23176-7_8-1

63 Haddon W. On the escape of tigers: an ecologic note. *Am J Public Heal Nations Heal* 1970;**60**:2229–34. doi:10.2105/AJPH.60.12.2229-b

64 Belin M-Å, Tillgren P. Vision Zero. How a policy innovation is dashed by interest conflicts, but may prevail in the end. *Scand J public Adm* 2013;**16**:83–102.

65 Bergh T, Remgård M, Carlsson A, *et al.* 2+1-roads Recent Swedish Capacity and Level-of-Service Experience. *Transp Res Procedia* 2016;**15**:331–45. doi:10.1016/j.trpro.2016.06.028

66 Johansson R. Vision Zero – Implementing a policy for traffic safety. *Saf Sci* 2009;**47**:826–31. doi:10.1016/j.ssci.2008.10.023

67 D'Elia A, Newstead S, Cameron M. Overall impact during 2001–2004 of Victorian speed-related package. Clayton, Victoria: : Monash University Accident Research Centre 2007.

68 Tingvall C, Michael J, Larsson P, *et al.* Saving Lives Beyond 2020: The Next Steps. In: *The Vision Zero Handbook*. Cham: : Springer International Publishing 2022. 1–52. doi:10.1007/978-3-030-23176-7_48-1

69 Safarpour H, Khorasani-Zavareh D, Soori H, *et al.* The Challenges of Vision Zero Implementation in Iran: A Qualitative Study. *Front Futur Transp* 2022;**3**:884930. doi:10.3389/ffutr.2022.884930

70 Abebe HG. Road Safety Policy in Addis Ababa: A Vision Zero Perspective. *Sustainability* 2022;**14**:5318. doi:10.3390/su14095318

71 Ahmadi N. Vision Zero. In: *Casebook of Traumatic Injury Prevention*. Cham: :

Springer International Publishing 2020. 285–300. doi:10.1007/978-3-030-27419-1_19
72 Thamarangsi T. The "Triangle That Moves the Mountain" and Thai Alcohol Policy Development: Four Case Studies. *Contemp Drug Probl* 2009;**36**:245–81. doi:10.1177/009145090903600112
73 Carroll A, Wood L, S Tantivess. Many Things to Many People—A Review of ThaiHealth. *ihppthaigov.net* Published Online First: 2007.http://www.ihppthaigov.net/DB/publication/attachresearch/230/chapter1.pdf (accessed 19 Oct 2022).
74 Sopitarchasak S, Adulyanon S, Lorthong T. Thai Health Promotion Foundation: Innovative Enabler for Health Promotion. *World Health Popul* 2015;**16**:62–71. doi:10.12927/whp.2015.24316
75 Adulyanon S. Funding health promotion and disease prevention programmes: an innovative financing experience from Thailand. *WHO South-East Asia J Public Heal* 2012;**1**:201. doi:10.4103/2224-3151.206932
76 Buasai S, Kanchanachitra C, Siwaraksa P. The way forward: experiences of health promotion development in Thailand. *Promot Educ* 2007;**14**:250–3. doi:10.1177/10253823070140041301
77 Witvorapong N, Ratisukpimol W, Watanapongvanich S. Effectiveness of alcohol-prevention social marketing in the presence of alcohol advertising. *J Soc Mark* 2019;**9**:309–28. doi:10.1108/JSOCM-01-2018-0003
78 Chandoevwit W, Thampanishvong K. TDRI QUARTERLY REVIEW. Published Online First: 2014.http://tdri.or.th/wp-content/uploads/2014/09/TDRI+QR-june-web-preview.pdf (accessed 19 Oct 2022).
79 Hamann SL, Mock J, Hense S, *et al.* Building tobacco control research in Thailand: Meeting the need for innovative change in Asia. *Heal Res Policy Syst* 2012;**10**. doi:10.1186/1478-4505-10-3
80 Suriyawongpaisal P, Patanavanich R, Aekplakorn W, *et al.* Paradox of sustainability in tobacco control in Thailand: A comprehensive assessment of three-decade experiences. *Int J Health Plann Manage* 2021;**36**:381–98. doi:10.1002/HPM.3089
81 Pongutta S, R Suphanchaimat. Lessons from the Thai health promotion Foundation. *ncbi.nlm.nih.gov* 2019.
82 Thamarangsi T. Addiction research centres and the nurturing of creativity. *Addiction* 2013;**108**:1201–6. doi:10.1111/j.1360-0443.2012.03795.x
83 Laslett A-M, Room R, Waleewong O, *et al. Harm to others from drinking: Patterns in nine societies*. 2019. https://apps.who.int/iris/handle/10665/329393?locale-attribute=ar&order=desc&scope=&query=harm to others&sort_by=score&rpp=10&search-result=true (accessed 19 Oct 2022).
84 Preampruchcha P, Suwanno N, Petchana B, *et al.* The effects of others' drinking on the harms to children in Thailand: Lessons from the WHO-ThaiHealth project. *PLoS One* 2022;**17**. doi:10.1371/JOURNAL.PONE.0265641
85 Health SA-WS-EAJ of P. The need for innovative health promotion financing system in Thailand. *apps.who.int* Published Online First: 2012.https://apps.who.int/iris/bitstream/handle/10665/206122/B4854.pdf?sequence=1#page=82 (accessed 19 Oct 2022).
86 Organ, O Waleewong. Moving Thailand's mountain of alcohol-related harm. *Bull World Health Organ* 2017;**95**:487–8. doi:10.2471/BLT.17.020717
87 Charoenca N, Mock J, Kungskulniti N, *et al.* Success Counteracting Tobacco Company Interference in Thailand: An Example of FCTC Implementation for Low- and Middle-income Countries. *Int J Environ Res Public Health* 2012;**9**:1111–34. doi:10.3390/ijerph9041111

88 Patanavanich R, Control SG-T. Successful countering of tobacco industry efforts to overturn Thailand's ENDS ban. *tobaccocontrol.bmj.com* 2020;**0**:1–10. doi:10.1136/tobaccocontrol-2020-056058
